# Exosomes Derived from Stem Cells from the Apical Papilla Promote Dentine-Pulp Complex Regeneration by Inducing Specific Dentinogenesis

**DOI:** 10.1155/2020/5816723

**Published:** 2020-05-27

**Authors:** Xueying Zhuang, Lingli Ji, Huan Jiang, Yao Liu, Xuemei Liu, Jing Bi, Weidong Zhao, Zhenjiang Ding, Xu Chen

**Affiliations:** ^1^Department of Pediatric Dentistry, School and Hospital of Stomatology, China Medical University, Shenyang 110002, China; ^2^Liaoning Provincial Key Laboratory of Oral Diseases, Shenyang 110002, China; ^3^Department of Developmental Cell Biology, School of Life Sciences, China Medical University, Shenyang 110122, China

## Abstract

Regenerative endodontic procedures (REPs) are a new option for the treatment of dental pulp or periapical diseases in permanent teeth with open apices. Histologically, the new tissues formed in the root canal after REPs are mainly cementum- or bone-like mineralised tissues, but not the real dentine-pulp complex. Therefore, how to promote dentine-pulp complex regeneration and improve the clinical effects of REPs has become a prominent research topic. Stem cells from apical papilla (SCAP) are derived from the dental papilla that can differentiate into primary odontoblasts and dental pulp cells that produce root dentine and dental pulp. Exosomes are the key regulator for the paracrine activity of stem cells and can influence the function of recipient cells. In this study, SCAP-derived exosomes (SCAP-Exo) were introduced into the root fragment containing bone marrow mesenchymal stem cells (BMMSCs) and transplanted subcutaneously into immunodeficient mice. We observed that dental pulp-like tissues were present and the newly formed dentine was deposited onto the existing dentine in the root canal. Afterwards, the effects of SCAP-Exo on the dentinogenesis of BMMSCs were elucidated *in vitro*. We found that the gene and protein expression of dentine sialophosphoprotein and mineralised nodule formation in BMMSCs treated with SCAP-Exo were significantly increased. In summary, SCAP-Exo were endocytosed by BMMSCs and obviously improved their specific dentinogenesis. The use of exosomes derived from dental stem cells could comprise a potential therapeutic approach for dentine-pulp complex regeneration in REPs.

## 1. Introduction

The treatment of dental pulp or periapical diseases in permanent teeth with open apices presents challenges to clinicians. The traditional method of apexification is restricted by the incomplete root formation with the open apex and short root. Regenerative endodontic procedures (REPs), a new treatment option for immature teeth with necrotic pulp and/or periapical periodontitis, are a biological method based on the concept of the triad of tissue engineering [1]. The tissue regeneration in REPs is mainly due to the cell-homing effect, which is directed by autogenous dental stem cells recruited into the empty root canal, such as residual dental pulp stem cells, stem cells from apical papilla (SCAP), periodontal ligament stem cells, and bone marrow mesenchymal stem cells (BMMSCs) from the jaw bone [2, 3]. REPs allow for the elimination of symptoms and bony healing and result in continued root development with increased root wall thickness and increased root length. However, our previous study showed that free cellular cementum-like tissues and bone-like mineralised structures are formed after REPs in the teeth of beagles with periapical periodontitis [4]. Studies have also indicated that the newly formed tissues in the canal after REPs are not real dentine-pulp complex but rather heterogeneous tissues including connective tissue, cementum-like tissue, and bone-like tissue [5, 6]. Further, the survival of dental stem cells in cases of pulp necrosis and apical periodontitis is not sufficient, and BMMSCs from the jaw bone might be the main stem cells that are homing to the root canal during REPs [7]. Therefore, how to promote dentine-pulp complex regeneration and improve the clinical effects of REPs have become a prominent research topic.

Following the development of tissue engineering, recent evidence has indicated that the tissue regeneration based on mesenchymal stem cells (MSCs) is mainly dependent on paracrine effects such as the secretion of trophic factors, cytokines, and extracellular vesicles [8]. Exosomes derived from mesenchymal stem cells (MSC-Exo) are extracellular microvesicles with a cup-shaped double-membrane structure and a size of 30–150 nm in diameter [9, 10]. Further, MSC-Exo contain growth factors; are rich in bioactive lipids, proteins, mRNAs, and regulatory miRNA; play important roles in cell-cell communication; and can influence the function of recipient cells [11, 12]. MSC-Exo serve as paracrine or autocrine mediators that regulate tissue regeneration. To date, MSC-Exo have been applied extensively to tissue regeneration and restoration modalities such as alleviating traumatic brain injury, enhancing cardiac repair, protecting the kidney from acute ischemia-reperfusion injury, and promoting muscle regeneration, among other applications [13–16].

SCAP play a critical role in tooth development and pulp regeneration in permanent teeth with open apices. SCAP can differentiate into primary odontoblasts and dental pulp cells that produce root dentine and dental pulp, thus serving as a promising stem cell source for dental pulp regeneration. Moreover, exosomes derived from stem cells from the apical papilla (SCAP-Exo) might carry the specific biological information of SCAP. Thus, we hypothesized that SCAP-Exo administration during REPs might enhance dentinogenesis and improve dentine-pulp complex regeneration. In this study, we evaluated the potential of SCAP-Exo to facilitate dentine-pulp complex regeneration, as well as the possible regulatory effects of SCAP-Exo on the biological function of BMMSCs, especially with respect to dentinogenesis capacity.

## 2. Materials and Methods

### 2.1. SCAP Isolation and Identification

Human impacted third molar with immature roots were collected from each healthy patient (12–15 years old) in the Dental Clinics at the School of Stomatology affiliated with China Medical University. The study was approved by the Ethics Committee of the School of Stomatology, China Medical University (201508). The apical papilla was gently removed from the tooth, minced, and digested in a solution of 2 mg/mL collagenase type I (Worthington, USA) and 4 mg/mL dispase II (Mannheim, Germany). Single-cell suspensions were seeded in 10 cm culture dishes and cultured with an alpha minimum essential medium (*α*-MEM, HyClone, USA) supplemented with 15% fetal bovine serum (FBS, MRC BRL), 2 mM L-glutamine (Biosource/Invitrogen, USA), 100 U/mL penicillin-streptomycin (HyClone), and 0.1 mM L-ascorbic acid 2-phosphate (WAKO, Japan) and maintained in 5% CO_2_ at 37°C. SCAP were identified by flow cytometry using anti-CD29, CD44, CD105, CD146, CD34, and CD45 antibodies (Abcam, USA). The multipotent differentiation potentials of osteogenesis and adipogenesis of SCAP were evaluated after osteogenic and adipogenic induction for 4 weeks. Alizarin red S and Oil red O staining were used to detect the formation of mineralised nodules and lipid droplets. SCAP from the third passage were used in the experiment.

### 2.2. BMMSC Isolation and Identification

Six to eight weeks Wistar rats (purchased from Vitaliver, Beijing, China) were selected and anesthetized with 2% pentobarbital sodium. The femurs and tibia of the hind limbs were separated to expose the bone marrow cavity. Then, 100 U/mL penicillin-streptomycin and 2% BSA (Meilun, China) were used to rush out the bone marrow. Single-cell suspensions were seeded in 10 cm culture dishes and cultured with the conventional medium in 5% CO_2_ at 37°C. The multipotent differentiation potentials of osteogenesis and adipogenesis of BMMSCs were evaluated the same as SCAP.

### 2.3. SCAP-Exo Isolation and Identification

SCAP-Exo were isolated according to previous protocols [17, 18]. For exosome isolation, a conventional culture medium was replaced with a serum-free medium when cells reached 60–80% confluence, and SCAP were cultured for an additional 48 h. Then, the supernatant was centrifuged sequentially at 4°C 3,000 × *g* for 20 min, 20,000 × *g* for 30 min, and 120,000 × *g* for 2 h. Finally, the exosome pellets were resuspended in 200 *μ*L of PBS. 20 *μ*L SCAP-Exo was added to 30 *μ*L lysis buffer (Beyotime Biotech Co., Shanghai, China) for 1 h on ice. The total concentration of exosomes was detected by the bicinchoninic acid (BCA) kit (Beyotime, China). SCAP-Exo was stored at -80°C for subsequent experiments.

The morphology of SCAP-Exo was identified with a transmission electron microscope (H-800, Hitachi, Japan). The size of these exosomes was analysed by nanoparticle tracking analysis. Furthermore, CD9 and Alix were detected by western blot using specific antibodies against CD9 (1 : 250, Abcam, USA) and Alix (1 : 500, Abcam, USA).

### 2.4. Preparation of Tooth Fragments

The teeth were obtained from clinically healthy premolars extracted for orthodontic reasons. The periodontal tissue of the root surface was removed with scalpel blades, and then, the cementum, dental pulp tissue, predentine, and partial dentine were removed with a fissure bur. The diameter of root canal space was expanded to about 3 mm, and the teeth were split into 5 mm length root segments. Next, the tooth fragments were immersed in 17% EDTA for 5 min, washed in deionised water for 10 min in an ultrasonic cleaner, and then immersed in 5% EDTA for 10 min, which was followed by washing in deionised water for 10 min in an ultrasonic cleaner. One of the tooth fragment orifices was sealed with glass ionomer cement (Fuji IX, GC, Tokyo, Japan). Then, it was stored in phosphate-buffered solution supplemented with 50 U/mL penicillin and 50 mg/mL streptomycin at 4°C.

### 2.5. Animal Experiments

This study was approved by the Ethics Committee of China Medical University for animal experiments (201315). All operations were performed on 6-week-old nude mice (*n* =10) under general anaesthesia. First, 50 *μ*g/mL SCAP-Exo and a 4 × 10^5^ BMMSCs with gelatine sponge (Xiang En, Jiang Xi, China) were introduced into the tooth fragments and transplanted subcutaneously into the backs of immunodeficient mice; 4 × 10^5^ BMMSCs with gelatine sponge were inserted into the tooth fragments as control. Each mouse received four tooth fragments, two on each side. There were 10 samples in each group. Twelve weeks after transplantation, the mice were euthanised and the tooth fragments were taken out for histological analysis.

### 2.6. Histological Examination

Samples were fixed in 4% paraformaldehyde for 24 h and decalcified in 10% EDTA (pH 7.4, MeilunBio, China) for 3 months, and then, all samples were embedded in paraffin and cut into 5 *μ*m sections. For histological analysis, sections were stained with H&E solution. Next, we counted the number of odontoblasts and measured the thickness of the new dentine using ImageJ software (1.50i, National Institutes of Health, Bethesda, MD, USA).

### 2.7. Exosomes Uptake Assay

SCAP-Exo were labelled using a PKH-26 Red Fluorescent Cell Linker Kit (Sigma, USA), as per the manufacturer's recommended protocol. Briefly, SCAP-Exo (300 *μ*g protein equivalent of exosomes in 250 *μ*L Diluent C) were stained with PKH26 (1 *μ*L of the PKH26 ethanolic dye solution in 250 *μ*L Diluent C) for 4 min at room temperature by pipetting. Then, 500 *μ*L of exosome-depleted serum was added to terminate the labelling reaction. Next, 2 × 10^4^ BMMSCs were seeded on glass coverslips and placed inside a 12-well plate. Twenty-four hours after seeding, PKH-26-labelled SCAP-Exo were incubated with BMMSCs for 3 h. An equal volume of PBS was added to the control group. Cells were fixed with 4% paraformaldehyde, and the nuclei were stained with DAPI. Finally, we determined whether SCAP-Exo were internalised by BMMSCs using a fluorescent microscope.

### 2.8. Cell Counting Kit-8 (CCK-8) Assay

To assess the effect of SCAP-Exo on cell proliferation, BMMSCs were incubated with a medium supplemented with different concentrations of SCAP-Exo and proliferation was determined using a CCK-8 kit (Dojindo, Kumamoto, Japan). In brief, BMMSCs were seeded in 96-well plates (2,000 cells/well) and cultured with a conditioned medium (containing 0, 5, 20, and 50 *μ*g/mL SCAP-Exo). After 1, 3, and 5 days, approximately 10 *μ*L of CCK-8 solution with 90 *μ*L of *α*-MEM was added to each well, and the plate was incubated for another 2 h at 37°C. The optical density was detected with a microplate reader (Tecan, Austria) at the wavelength of 450 nm.

### 2.9. Ki-67 Staining Assay

BMMSCs (2 × 10^4^/well) were seeded on glass coverslips placed inside a 12-well plate and cultured for 48 h. Cells were fixed with 4% paraformaldehyde and permeabilised using 0.3% triton X-100 (Beyotime, China). Then, glass coverslips were incubated with an anti-Ki-67 (CST, USA) primary antibody at 4°C overnight. Next, samples were incubated with the fluorescent secondary antibody (Proteintech, Chicago, USA) at room temperature for 2 h; DAPI staining was then performed, and antiquench sealed tablets were used. Ki-67-positive and total cell numbers were counted based on 10 images per sample using a fluorescence microscope. The number of Ki-67-positive cells was indicated as a percentage of the total cell number.

### 2.10. In Vitro Osteo-/Odontogenic Differentiation Assay

BMMSCs (1 × 10^5^ cells/well) were seeded in 6-well plates and pretreated with a conditioned medium containing different doses of SCAP-Exo (0, 5, 20, and 50 *μ*g/mL) for 4 days. Then, culture medium was replaced with an osteo-/dentinogenic induction medium containing 1.8 mM monopotassium phosphate (Sigma-Aldrich) and 10 nM dexamethasone (Sigma-Aldrich). After incubation for 7 days, total mRNA was extracted, and 10 days later, proteins were isolated to assess the expression level of an osteogenic/dentinogenic marker.

### 2.11. Real-Time Polymerase Chain Reaction (PCR) Analysis

Real-time PCR was performed 30 cycles using the GoTaq® qPCR Master Mix (Promega Corporation, Madison, WI, USA) in a 7500 real-time PCR system (Applied Biosystems). The mRNA expression levels of dentin sialophosphoprotein (*DSPP*), alkaline phosphatase (*ALP*), and runt-related transcription factor 2 (*Runx2*) were evaluated. Glyceraldehyde-3-phosphate dehydrogenase (*GAPDH*) was used as a housekeeping gene for normalisation. The data were calculated and statistically analysed using the formula 2^−*ΔΔ*Ct^. The sequences of primers were as follows: *DSPP* forward primer, 5′-CTGTTGGGAAGAGCCAAGATAAG-3′; *DSPP* reverse primer, 5′-CCAAGATCATTCCATGTTGTCCT-3′; *ALP* forward primer, 5′-TAAGGACATCGCCTACCAGCTC-3′; *ALP* reverse primer, 5′-TCTTCCAGGTGTCAACGAGGT-3′; *Runx2* forward primer, 5′-GCACCCAGCCCATAATAGA-3′; *Runx2* reverse primer, 5′-TTGGAGCAAGGAGAACCC-3′; *GAPDH* forward primer, 5′-CCGGCGTCCGACCTGTGAAC-3′; *GAPDH* reverse primer, 5′-GGGCGAAGGCTCCAGAGGA-3′.

### 2.12. Western Blot Analysis

Total protein was extracted using lysis buffer (Beyotime Biotech Co., Shanghai, China). 20 *μ*g of whole protein was loaded and separated by 12% SDS-polyacrylamide gel electrophoresis and transferred electrophoretically to a polyvinylidene difluoride membrane (Millipore Corporation, USA). The membrane was blocked by incubation in 4% BSA for 1 h at room temperature and then incubated with anti-DSPP (1 : 500, Santa Cruz Biotechnology, USA), anti-Runx2 (1 : 250, Santa Cruz Biotechnology, USA), anti-ALP (1 : 200, Santa Cruz Biotechnology), or anti-*β*-actin (1 : 1,000, Santa Cruz Biotechnology) primary antibodies at 4°C overnight. Goat anti-rabbit/anti-mouse IgG IRDyel 800cw secondary antibody (1 : 1,000, Abbkine, USA) was used to incubate the membranes for 1 h at room temperature. The protein bands were detected with an Odyssey CLx instrument (LI-COR, Lincoln, NE, USA), and grayscale analysis was performed with ImageJ software (1.50i, National Institutes of Health, Bethesda, MA, USA).

### 2.13. Alizarin Red S Staining

After culturing with an osteo-/odontogenic induction medium for 3 weeks, all the cultures were fixed with 60% isopropanol for 1 min. Alizarin red S solution was added to each well for staining. Nonspecific staining was removed by repeated washing with distilled water. Semiquantitative analysis of mineralised nodule formation was performed with ImageJ software.

### 2.14. Statistical Analysis

Statistical analysis was performed using SPSS 20.0 software (SPSS Inc., Chicago, IL, USA). All data were recorded as the mean ± SD and replicated for three independent experiments. The differences were tested by one-way analysis of variance (ANOVA). The differences were considered significant if the *P* value was less than 0.05.

## 3. Results

### 3.1. Identification of SCAP, BMMSCs, and SCAP-Exo

The majority of isolated SCAP retained a spindle shape and formed colonies in primary culture (Fig. S1A). When SCAP were cultured in an osteogenic- and adipogenic-conditioned medium for 4 weeks, SCAP were found to form mineralised nodules based on Alizarin red S staining (Fig. S1B) and lipid droplets based on staining with Oil red O (Fig. S1C). Moreover, flow cytometric analysis showed that SCAP expressed mesenchymal stem cell surface markers including CD29, CD44, CD105, and CD146 but failed to express the haematopoietic markers CD34 and CD45 (Fig. S1D).

When BMMSCs were cultured for 7 days, cell adherent growth was observed by the microscope, showing the short spindle or polygon shape (Fig. S2A). After cultured with an osteogenic- or adipogenic-conditioned medium for 3 weeks, BMMSCs were also found to form mineralised nodules and lipid droplets (Fig. S2B, 2C).

**Figure 1:**
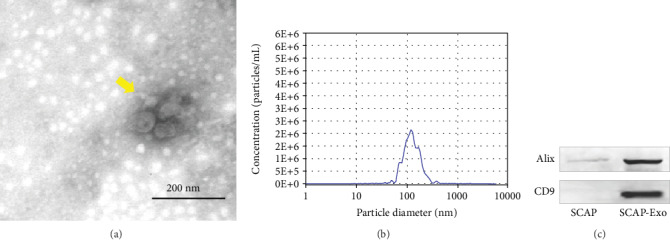
Identification of exosomes from stem cells of the apical papilla (SCAP-Exo). (a) Morphology of SCAP-Exo (yellow arrow) based on transmission electron microscopy. (b) Size distribution of particles in the pellet as measured by nanoparticle tracking analysis. (c) Western blot analysis showing that SCAP-Exo were positive for the exosomal-specific markers CD9 and Alix.

By transmission electron microscopy, SCAP-Exo were observed to contain a bilayer membrane and cup-plate-shaped structures (Figure 1(a)). Moreover, nanoparticle tracking analysis showed a major peak in particle size at 120.6 nm (Figure 1(b)). Furthermore, SCAP-Exo expressed the specific exosomal markers CD9 and Alix (Figure 1(c)) based on western blot.

### 3.2. SCAP-Exo Promoted BMMSC-Based Dentine-Pulp Complex Regeneration

**Figure 2:**
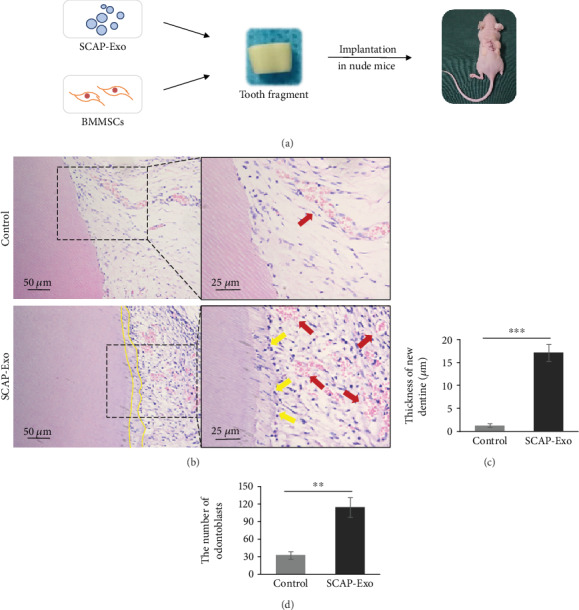
Exosomes from the stem cells of the apical papilla (SCAP-Exo) promoted the regeneration of the dentine-pulp complex *in vivo*. (a) Schematic diagram of the animal experiment: SCAP-Exo, bone marrow mesenchymal stem cells (BMMSCs), and gelatine sponges as scaffolds were inserted into the canal of the tooth fragments, while the control group was treated with the same preparation without SCAP-Exo. The tooth fragments were then implanted into the nude mice. (b) HE staining showed a newly continuous layer of dentine (dotted line), odontoblast-like cells with overt polarised morphology (yellow arrow), and enhanced vascular formation (red arrow) in the experimental group. (c, d) The thickness of the new dentine and the number of odontoblasts were higher in the SCAP-Exo group than that in the control group (^∗∗^*P* < 0.01, ^∗∗∗^*P* < 0.001, *n* = 10). Error bars indicate means ± SD.

As shown in the schematic diagram (Figure 2(a)), tooth fragments with SCAP-Exo, BMMSCs, and scaffolds were implanted subcutaneously into immunodeficient mice, whereas the control group was treated with the same preparation without SCAP-Exo. After 12 weeks, histological analysis showed that a new continuous dentine layer was formed in the SCAP-Exo group, in which the number of odontoblasts (yellow arrows) was significantly increased, with a high columnar shape and polarised morphology. They were located at the junction of pulp and predentine in an ordered arrangement, forming an odontoblast process into the dentinal tubules. In addition, more vascular lumens (red arrow) were also observed. In the control group, we did not observe the formation of this new dentine and odontoblast layer (Figure 2(b)). Both the thickness of the new dentine and the number of odontoblasts were higher in the SCAP-Exo group than that in the control group (Figures 2(c) and 2(d)). These data indicated that SCAP-Exo promoted BMMSC-based dentine-pulp complex regeneration.

### 3.3. SCAP-Exo Were Endocytosed by BMMSCs

**Figure 3:**
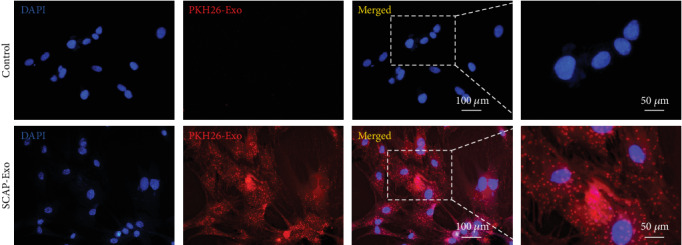
Exosomes from the stem cells of the apical papilla (SCAP-Exo) were endocytosed by bone marrow mesenchymal stem cells (BMMSCs). PKH-26-labelled SCAP-Exo (red) was internalised into the cytoplasm of DAPI-labelled BMMSCs (blue). In the negative control group of BMMSCs without exosomes, only the nuclei of BMMSCs were stained with DAPI (blue).

We next added PKH-26-labelled SCAP-Exo into the culture media of BMMSCs *in vitro*. After 3 h, fluorescent staining showed that many SCAP-Exo (red) were located in the cytoplasm of DAPI-labelled BMMSCs (blue); however, only the nuclei of BMMSCs were stained with DAPI (blue) in the control group (Figure 3). These data suggested that BMMSCs could take up SCAP-Exo *via* endocytosis.

### 3.4. SCAP-Exo Induced the Dentinogenesis of BMMSCs

**Figure 4:**
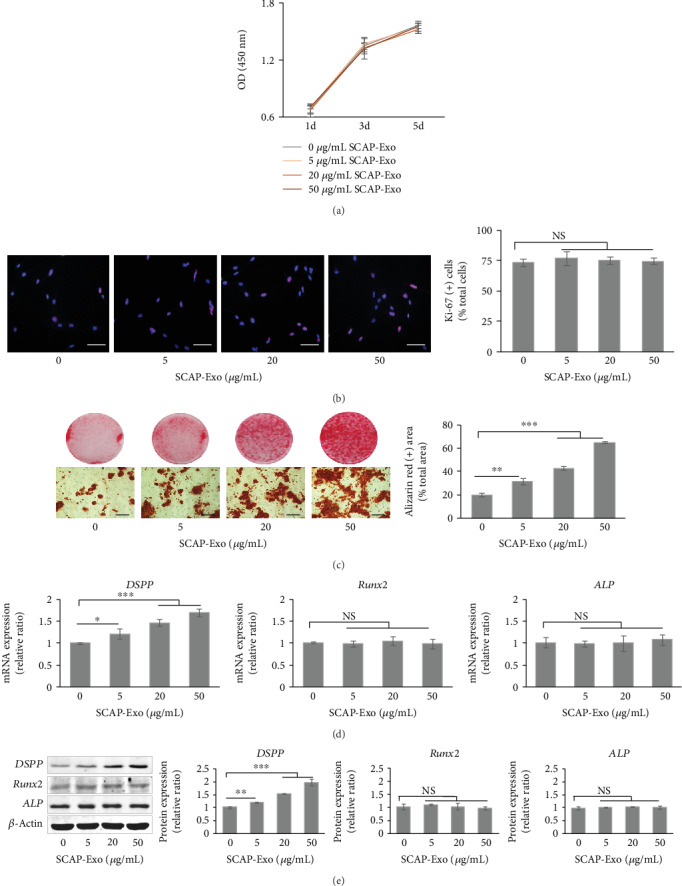
Exosomes from stem cells of the apical papilla (SCAP-Exo) induced the odontogenic differentiation of bone marrow mesenchymal stem cells (BMMSCs). (a, b) CCK-8 and Ki-67 assays showed that SCAP-Exo had no significant effect on the proliferation of BMMSCs. Scale bars = 100 *μ*m. (c) Alizarin red S staining showed that SCAP-Exo could increase mineralised nodule formation in a dose-dependent manner. Scale bars = 100 *μ*m. (d) Real-time PCR analysis revealed that SCAP-Exo could augment *DSPP* mRNA expression levels in BMMSCs without exerting effects on *Runx2* and *ALP* mRNA expression levels. (e) Western blot analysis indicated that the protein expression levels of ALP and Runx2 were not significantly changed, but that high doses (20 and 50 *μ*g/mL) of SCAP-Exo significantly improved the protein expression levels of DSPP (^∗^*P* < 0.05, ^∗∗^*P* < 0.01, and ^∗∗∗^*P* < 0.001, NS = not significant, *n* = 3). Error bars indicate means ± SD.

CCK-8 assays and Ki-67 staining showed that SCAP-Exo exerted no significant effects on the proliferation rate of BMMSCs (Figures 4(a) and 4(b)). To further explore whether and how SCAP-Exo regulate the osteo-/odontogenic differentiation of BMMSCs, we used SCAP-Exo to treat BMMSCs and then cultured cells under osteogenic induction conditions. Alizarin red S staining showed that mineralised nodule formation was increased markedly with SCAP-Exo treatment in a concentration-dependent manner (Figure 4(c)). Furthermore, we examined the expression levels of osteo-/odontogenic genes and proteins. As shown in Figure 4(d), SCAP-Exo increased the gene expression of the dentinogenic marker *DSPP*, and the 20 and 50 *μ*g/mL SCAP-Exo groups exhibited a more robust increase compared to that in the control group (*P* < 0.001). Nevertheless, SCAP-Exo had no significant effects on the gene expression levels of *ALP* and *Runx2*. Consistent with qPCR analysis, the protein expression of DSPP also increased obviously upon 20 *μ*g/mL and 50 *μ*g/mL SCAP-Exo treatment (*P* < 0.001); however, SCAP-Exo also did not affect protein levels of ALP and Runx2 (*P* > 0.05; Figure 4(e)). Therefore, these data indicated that SCAP-Exo promoted the specific dentinogenesis of BMMSCs.

## 4. Discussion

Strategies for dentine-pulp complex regeneration are conventionally categorised into cell-based and cell-free therapies. Cell-based therapies consist of transplanting exogenous stem cells, loaded onto scaffolds incorporated with signalling molecules, into the root canal system of the host to allow regeneration. The modes of MSC-based therapy in tissue regeneration consist of MSCs mobilised to the site and differentiated into functional cells to replace damaged cells [19]. In general, dental-derived MSCs are selected for transplantation into the root canal. Xuan *et al.* have reported that human deciduous DPSCs are able to regenerate whole dental pulp and form new dentine to promote root development [20], while SCAP is derived from the immature tissue of the apical papilla and is composed of more undifferentiated cells than dental pulp [21]. However, the clinical translational application of MSCs is limited by ethical issues, availability, stem cell isolation and storage, and professional technical skills, among other factors. Additionally, increasing evidence has indicated that most exogenous MSCs quickly disappear posttransplant [22]. Based on “cell homing” theory, the cell-free approach achieves tissue regeneration by modulating the migration, proliferation, and differentiation of endogenous stem cells residing around the root apex. It is believed that “cell homing” is the basis for the majority of current clinical approaches to REPs [23]. Some scholars have suggested that cell niches for stem cell populations could establish a suitable microenvironment by producing immunoregulatory factors and promoting paracrine activities to stimulate the differentiation of progenitor cells *in situ* [19, 24, 25]. Natural compounds or bioactive substances have been shown to have the potential to induce specific dentinogenesis [26, 27].

Currently, the approach based on MSC-Exo provides new therapeutic alternatives for tissue regeneration. It has been shown that exosome characteristics differ in diverse cell types [28]. Further, the complex composition of MSC-Exo might mirror that of their parental cells and their ability to migrate towards specific tissue [22]. MSC-Exo also contain numerous bioactive molecules that can be transferred to target cells to influence their fate and tissue regeneration. A previous study indicated that exosomes isolated from dental pulp stem cells could induce the odontogenic differentiation *in vitro* and trigger the regeneration of dental pulp-like tissue *in vivo* [27]. In this study, we evaluated the potential of SCAP-Exo to promote dentine-pulp complex regeneration. Our *in vivo* study indicated that SCAP-Exo could facilitate the generation of the newly formed dentine tissues and odontoblasts. Furthermore, there were a large number of polar odontoblasts in an ordered arrangement. Odontoblast polarisation marks the morphological change from symmetrical mesenchymal cell to asymmetrical odontoblast, which is a prerequisite and fundamental step for tooth development and dentine formation [29]. In addition, we observed numerous blood vessels in newly formed pulp-dentine complex induced by SCAP-Exo in the root canal. Angiogenesis is a prerequisite for tissue regeneration through the provision of sufficient oxygen and nutrients for tissue survival [30]. It has been reported that dental pulp stem cell-derived exosomes had an effect on angiogenesis [31]. SCAP can secrete a large number of proangiogenic factors [32]. There is a need to clarify whether SCAP-Exo can promote angiogenesis or not and its related mechanism in the future. Summarily, the histologic findings suggested that SCAP-Exo could improve the dentine-pulp complex regeneration in REPs.

To explore the underlying mechanisms through which SCAP-Exo promote the regeneration of pulp-dentine complex, we evaluated their effects on the proliferation and differentiation of BMMSCs *in vitro*. We found that SCAP-Exo did not affect BMMSC proliferation but significantly regulated their dentinogenesis by enhancing DSPP expression in BMMSCs, as well as the formation of mineralised nodules. DSPP, a precursor protein, can be cleaved into two proteins, specifically dentine sialoprotein (DSP) and dentine phosphoprotein (DPP), which are believed to play a crucial role in dentine formation [33]. DSPP is secreted by odontoblasts and is the most abundant in the extracellular matrix; moreover, it can serve as a template for mineral nucleation and growth to mediate the mineralisation of extracellular matrix during dentine formation [34]. In addition, it is a terminal differentiation marker of odontoblasts [35]. Recently, Frozoni used BMMSCs in pulp cap in a mouse model and they found that GFP labelled-BMMSCs were directly utilised and GFP-labelled DSP positive odontoblasts were observed in the root canal 7 weeks later [36]. This study may shed some light on how to explain their observations. The endogenous dental pulp stem cells around the pulp exposure site might transfer specific substances into the transplanted BMMSCs and induce dentinogenesis of BMMSCs *via* a paracrine effect. In particular, in this study, SCAP-Exo treatment did not significantly influence the expression levels of another two mineralisation-related proteins, ALP and Runx2. We speculated that SCAP-Exo might mainly promote the secretion of DSPP rather than the differentiation of BMMSCs.

At present, how exosomes influence recipient cells remain elusive. Exosomes contain various RNAs (including mRNA, miRNA, and tRNA) and proteins [10, 37]. Baglio *et al*. showed that the five most abundant miRNAs in MSC-Exo account for a high proportion (50%) of the total miRNA, which indicates that specific miRNAs present in large amounts might have a physiological impact on target cells [38]. In addition, exosomal proteins could be directly utilised by recipient cells. Our previous study showed that recipient MSCs can receive exosomes from donor MSCs and reuse Fas derived from these exosomes to improve recipient MSC function [39]. In this study, we considered that SCAP-Exo might transfer the biological information of periapical tissue to reprogram the function of BMMSCs. However, the detailed mechanisms through which SCAP-Exo influence the biological function of BMMSCs need to be further elucidated.

SCAP-Exo serve as systemic cell-cell communication mediators that play an important role in tissue regeneration. We believed that exosomes released by various cell types in different states might contain diverse contents to deliver specific information, which could have different implications for the target cells. The application of SCAP-Exo could thus provide a new strategy to promote dentine-pulp complex regeneration after REPs. Further studies will need to focus on establishing an animal model of periapical periodontitis in permanent teeth with open apices to determine the efficacy of SCAP-Exo in REPs. However, it is crucial to select a suitable scaffold for carrying exosomes to the root canal. There is evidence that the geometrical and mechanical properties of scaffolds can influence cell behaviour and the adhesion of MSCs to the scaffold surfaces [40–42]. Further studies are needed to select suitable scaffold materials for future use of SCAP-Exo in the root canal and to elucidate the possible interaction between SCAP-Exo and scaffold materials during REPs as well.

## 5. Conclusions

SCAP-Exo could be endocytosed by BMMSCs, obviously improved their specific dentinogenesis, and promoted dentine-pulp complex regeneration. This study provided an optimised method for cell homing-based REPs in the clinic. It could be a promising therapeutic approach that uses standardised exosomal reagents in regenerative endodontics.

## Data Availability

The data used to support the findings of this study are available from the corresponding author upon request.

## Supplementary Materials

Supplementary Materials Supplementary Figure 1: identification of stem cells of apical papilla (SCAP). (A) SCAP formed colony-forming units in primary culture. (B) Alizarin red S staining showed the formation of mineralised nodules. (C) Oil red O staining showed lipid droplet formation. (D) Flow cytometric analysis showed that SCAP were positive for CD29, CD44, CD105, and CD146 but negative for CD34 and CD45. Supplementary Figure 2: identification of bone marrow mesenchymal stem cells (BMMSCs). (A) The morphology of BMMSCs. (B) Alizarin red S staining showed the formation of mineralised nodules. (C) Oil red O staining showed lipid droplet formation.
