# Supplementary material for: Exosomes Derived from Stem Cells from the Apical Papilla Promote Dentine-Pulp Complex Regeneration by Inducing Specific Dentinogenesis
Source: Stem Cells Int. 2020 May 27;2020:5816723. doi: 10.1155/2020/5816723 (PMC7273441; doi:10.1155/2020/5816723)
Supplement: Supplementary Materials — Supplementary Figure 1: identification of stem cells of apical papilla (SCAP). (A) SCAP formed colony-forming units in primary culture. (B) Alizarin red S staining showed the formation of mineralised nodules. (C) Oil red O staining showed lipid droplet formation. (D) Flow cytometric analysis showed that SCAP were positive for CD29, CD44, CD105, and CD146 but negative for CD34 and CD45. Supplementary Figure 2: identification of bone marrow mesenchymal stem cells (BMMSCs). (A) The morphology of BMMSCs. (B) Alizarin red S staining showed the formation of mineralised nodules. (C) Oil red O staining showed lipid droplet formation. [file 5816723.f1.zip › Supplementary Figure 1.pptx]

## Slide 1
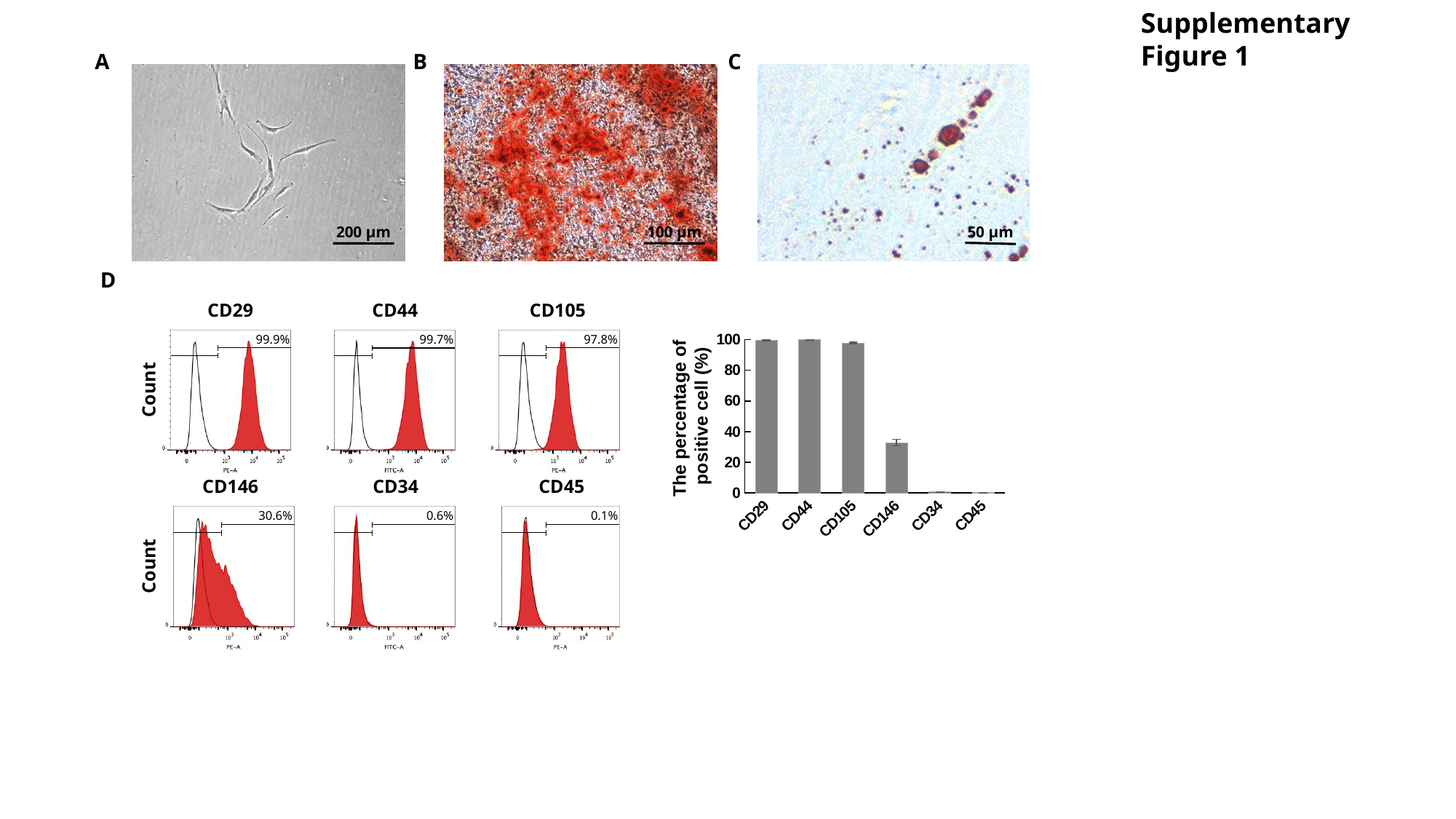

Supplementary Figure 1
A
50 μm
200 μm
B
C
100 μm
 D
CD29
CD44
CD105
Count
CD146
CD34
CD45
Count
99.9%
99.7%
97.8%
### Chart
| Category | |
|---|---|
| CD29 | 99.4 |
| CD44 | 99.8 |
| CD105 | 97.8 |
| CD146 | 32.7666666666667 |
| CD34 | 0.566666666666667 |
| CD45 | 0.133333333333333 |The percentage of
positive cell (%)
0.1%
30.6%
0.6%
